# Searching for “Environmentally-Benign” Antifouling Biocides

**DOI:** 10.3390/ijms15069255

**Published:** 2014-05-26

**Authors:** Yan Ting Cui, Serena L. M. Teo, Wai Leong, Christina L. L. Chai

**Affiliations:** 1 Department of Pharmacy, National University of Singapore, 18 Science Drive 4, Singapore 117543, Singapore; E-Mail: a0098691@nus.edu.sg; 2 Tropical Marine Science Institute, National University of Singapore, 18 Kent Ridge Road, Singapore 119227, Singapore; E-Mails: tmsteolm@nus.edu.sg (S.L.M.T.); tmslw@nus.edu.sg (W.L.); 3 Institute of Chemical and Engineering Sciences, Agency for Science Technology and Research, 8 Biomedical Grove, Singapore 138665, Singapore

**Keywords:** environmentally benign, anti-fouling, BIOWIN™; ECOSAR™; persistence, bioaccumulation, toxicities, organic compounds

## Abstract

As the result of the ecological impacts from the use of tributyltins (TBT) in shipping, environmental legislation for the registration of chemicals for use in the environment has grown to a monumental challenge requiring product dossiers to include information on the environmental fate and behavior of any chemicals. Specifically, persistence, bioaccumulation and toxicity, collectively known as PBT, are properties of concern in the assessment of chemicals. However, existing measurements of PBT properties are a cumbersome and expensive process, and thus not applied in the early stages of the product discovery and development. Inexpensive methods for preliminary PBT screening would minimize risks arising with the subsequent registration of products. In this article, we evaluated the PBT properties of compounds reported to possess anti-fouling properties using QSAR (quantitative structure-activity relationship) prediction programs such as BIOWIN™ (a biodegradation probability program), KOWWIN™ (log octanol-water partition coefficient calculation program) and ECOSAR™ (Ecological Structure Activity Relationship Programme). The analyses identified some small (*M*_r_ < 400) synthetic and natural products as potential candidates for environmentally benign biocides. We aim to demonstrate that while these methods of estimation have limitations, when applied with discretion, they are powerful tools useful in the early stages of research for compound selection for further development as anti-foulants.

## 1. Introduction

Biofouling is a major problem for the shipping industry. The majority of existing anti-fouling management relies on toxic coatings on vessel hulls to prevent settlement of marine organisms. The toxicants are incorporated into marine paints and leach from the surface layers of the coatings through a variety of mechanisms depending on the type of paint matrix used. Until recently, organotins were the most effective of these compounds for anti-fouling management. However due to severe toxicity and ecological problems associated with organotins (or broadly known as tributyltins, TBTs), the International Maritime Organisation (IMO) has banned the application of TBT on all vessels since 2001 and requires TBT-free coatings since 2008 [1]. The phenomenon of imposex was among the first indications of the non-target effects of TBTs which was only observed when the TBTs in antifouling coatings gained market share. Although it is well-known that TBTs are highly toxic, the assumption based on laboratory studies suggests that TBTs degrade reasonably rapidly to non-toxic compounds in the presence of sunlight [2]. However in practice, UV degradation is not as facile as predicted due to the poor of penetration of sunlight in water and the propensity for many compounds to bind to sediments. In view of the large quantities of TBTs discharged into the oceans and due to their lipophilicity, their bioaccumulation in marine organisms can be very high. For example, in fish the bioconcentration factors occur between 100–1000 [3].

There is increasing recognition that there is a need to design and make “green” products for our everyday applications. The twelve principles of green chemistry advocated by Anastas and Warner [4] include the notion of “safer” chemical design *i.e.*, compounds that leave no harmful footprints and that biodegrade to innocuous products. In this context, a “green” chemical is one that has minimal toxicity to humans and vertebrates, no adverse effects on ecology and the environment, should not bioaccummulate and biomagnify, and thus should readily biodegrade into environmentally benign products. The notion of “non-toxic” antifouling compounds is a contradiction in terms as larvae that have been exposed to biologically active chemicals are unlikely to successfully settle and recruit. Thus, a better definition of an environmentally benign product pays attention to the fate and effects of compounds in the environment, with the aim to produce short-lived molecules that do not last long enough to incur any impact on non-target species and the ecosystem as a whole. The TBT experience highlights the severe economical and ecological consequences that result when compounds are used without consideration for environmental fate. Quite early in the TBT debate, Evans [5] highlighted potential issues with alternative solutions. Most tin-free antifouling coatings relied on augmenting copper with booster biocides which are predominantly organic compounds, but these may not constitute a safer chemical design of anti-foulants. Although copper (II) is naturally found in the marine environment (estimated to be 0.5–3 µg/L) [6], a much larger amount of copper from anti-fouling coatings is estimated to be released into the oceans. In some places, there are already restrictions on the use of copper due to the high sedimentation, the persistent nature of copper, high bioaccumulation in marine organisms and the observations that certain copper forms are toxic [6]. Booster biocides such as Irgarol 1051 have been found to be persistent, in addition to producing degradation products more toxic than the original parent compound [7].

Fundamentally, a biocide is toxic but it is highly undesirable that the key antifouling mechanism should lie in insidious biological effects such as mutagenicity or in endocrine-disruptive pathways. Beyond toxicity, for a biocide to be considered environmentally benign, it is essential to manage the fate of the potential anti-foulants (and their degradation products) in the marine environment. In this context, PBT (persistence-bioaccumulation-toxicity) considerations are key traits to be monitored for compounds and their degradation products. A long environmental half-life of a compound will render the compound persistent and this in turn exacerbates the problems of bioaccumulation and toxicity as the product gains market share [8].

Biodegradability—the propensity of the chemical to undergo biodegradation—is a property of the compound that determines its half-life. However, in practice, only biodegradation rates can be measured, and these vary depending on the enzymatic pathways present in the source of cultured bacteria at any one place and time. Measurements of biodegradation are difficult and expensive. There are few standardized methods and most are fraught with reproducibility problems. A number of quantitative structure-activity relationship (QSAR) models are available [9] and a critical analysis of the various approaches to predict aquatic aerobic biodegradation was recently published by Rucker and Kummerer [10]. These QSAR models provide a prediction for biodegradability and although fraught with assumptions of their own, may be useful as a guide to identifying antifouling compounds that are biodegradable.

In this article, we considered a suite of organic compounds that have been used commercially as anti-fouling agents as well as compounds reported to show anti-settlement activities against fouling organisms. We applied available QSAR models to evaluate how environmentally benign they are. The Biodegradation Probability Program (BIOWIN™ v4.10, http://www.epa.gov/oppt/exposure/pubs/episuite.htm) developed by Syracuse Research Cooperation jointly with the Environmental Protection Agency (EPA), was used to estimate biodegradation as it remains one of the most commonly used free software in the registration process for biocides. BIOWIN™ utilizes functional group contribution approaches in the estimation of aerobic biodegradation of organic compounds in the presence of microorganisms in the environment. Within BIOWIN™, there are six separate models utilizing linear probability model (BIOWIN™ 1), non linear probability model (BIOWIN™ 2), semi-quantitative prediction of ultimate and primary biodegradation rates using multiple linear regressions (BIOWIN™ 3 and 4 respectively), linear and non linear Ministry of International Trade and Industry (MITI) model (BIOWIN™ 5 and 6). The last two employed a training set from a large dataset of discrete organic chemicals. BIOWIN™ 7 is different to all the models above in that it estimates the probability of fast biodegradation under methanogenic anaerobic conditions.

Different researchers have used the predictions from a combination of different models to estimate biodegradability [9,11,12]. In practice, combining models has been shown to improve the accuracy of predictions [13]. In our study, we included two sets of criteria for comparison. The first requires that either BIOWIN™ 2 or 6 has values of >0.5 (non linear model prediction) and BIOWIN™ 3 has values of >2.2 (ultimate biodegradation timeframe prediction) for a compound to be readily biodegradable [9]. In the context of this article, this will be referred as prediction criteria A. The BIOWIN™ program also automatically generates a yes/no qualitative prediction of ready biodegradability (known herewith as prediction criteria B) using the criteria that both BIOWIN™ 3 (weeks or faster) and BIOWIN™ 5 (>0.5) need to be “weeks” or faster in order to be classified as compounds that are easily biodegraded.

Bioaccumulation was estimated using KOWWIN™ v1.68 developed by the U.S. EPA. Compounds are considered potentially bioaccumulative if the log *K*_ow_ (octanol-water partition coefficient) is 3 or more [6]. For compounds with metals/coordination bond, the respective ligand form without the metal is estimated. For partially ionised compounds, the un-dissociated log *K*_ow_ is considered while for fully ionised compounds, the log *K*_ow_ value of the ionic form is used. Finally, predictions of toxicity to aquatic organisms (fish, daphnids, and green algae) were made using Ecological Structure Activity Relationship programme (ECOSAR™) [14], and comparisons made to Model predictions were compared to experimental data wherever available.

## 2. Results and Discussion

Many organic compounds have been reported to possess “anti-fouling” properties. These are mainly from laboratory studies where anti-fouling activity is equated to anti-settlement activity or killing activity against fouling organisms as well as compounds that are anti-bacterial or prevent biofilm formation. The latter compounds were not considered in this article. We restricted our analyses to compounds with known activity against macrofouling organisms. Antifouling compounds can be further classified into two main categories—synthetic compounds and natural products.

### 2.1. Synthetic Compounds

#### 2.1.1. Known Agricultural Poisons as Anti-Fouling Agents

The key booster biocides currently in commercial use are summarized in Table 1. These compounds have been used in the agricultural industry e.g., Irgarol 1051 and Diuron are herbicides, while copper/zinc pyrithione is a microbiocide. The environmental fate and behaviour of some of these booster biocides have been reviewed by many [6,15] and this provides us with experimental information for comparison with data obtained from modeling. The molecular weights of these compounds range from 200–400, with an average molecular weight of 281; total polar surface area (tPSA) (metals are not included in the calculation of tPSA) between 20–60, with an average of 35; and log *K*_ow_ between −0.3 and 4, with an average of 2.27.

Under criteria A, chlorothalonil, dichlorofluanid, irgarol, 2,3,5,6-tetrachloro-4-sulfuronyl (TCMS) pyridine, 2-(thiocyanomethylthio)-benzothiazole (TCMTB), Diuron, 4,5-dichloro-2-(*n*-octyl)-3-(2*H*)-isothiazolone (DCOIT), and Zineb are flagged as not readily biodegraded. Criteria B predicts that all the biocides in Table 1 are not biodegradable.

**Table 1:** Characterization of booster biocides. *M*_r_ = molecular weight; tPSA = total polar surface area; Log *K*_ow_ = log of water/octanol partition; * Log *K*_ow_ values calculated using the log octanol-water partition coefficient calculation program KOWWIN™; ** Refers to calculations of the ligand (without the metal) for copper/zinc pyrithione and zineb. ** Log *K*_ow_ of the ligand (without the metal) was estimated for copper pyrithione. ** tPSA of the ligand (without the metals) was estimated for copper/zinc pyrithione and zineb. Using BIOWIN™ (a biodegradation probability program) models: 1-linear, 2-non-linear, 3-ultimate, 4-primary, 5-linear Ministry of International Trade and Industry (MITI), 6-non-linear MITI, 7-anaerobic; Criteria A-model 2 or 6 > 0.5 and model 3 > 2.2; Criteria B-model 3 “weeks” or faster and model 5 > 0.5. Ecological Structure Activity Relationship programme (ECOSAR™): F = fish, D = daphnid, A = green algae. Experimental data were obtained from Material Safety Data Sheets (MSDS) unless specified otherwise.

Booster biocides [CAS number]	Structure	*M*_r_	tPSA	Log *K*_ow_	BIOWIN™	ECOSAR™ acute toxicity (mg/L) (Exp. values, mg/L)	
Chlorothalonil [1897-45-6]	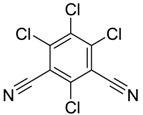	265.90	47.58	4.0 [15]	1: 0.51; 2: 0.61; 3: 1.62; 4: 2.67; 5: 0.09; 6: 0.004; 7: −0.77; A: NO B: NO	F: 6.98 (0.012) D: 4.62 (0.06) A: 6.50 (0.6)	
Dichlofluanid [1085-98-9]	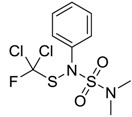	333.20	40.62	3.7 [15]	1: 0.31; 2: 0.005; 3: 1.93; 4: 3.02; 5: −0.15; 6: 0; 7: 0.05; A: NO B: NO	F: 62.02 (0.032) D: 37.63 A: 36.87	
Irgarol 1051 [28159-98-0]	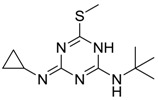	253.37	61.14	3.95 [15]	1: 0.44; 2: 0.09; 3: 2.43; 4: 3.33; 5: 0.08; 6: 0.02; 7: −0.02; A: NO B: NO	F: 33.65 (0.7) D: 20.73 (5.3) A: 21.62 (0.002)	
TCMS Pyridine [13108-52-6]	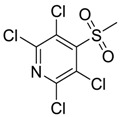	294.96	47.03	1.95 *	1: −0.28; 2: 0; 3: 1.51; 4: 2.74; 5: −0.17; 6: 0.0007; 7: −0.21; A: NO B: NO	F: 267.43 D: 151.18 A: 110.58	
TCMTB [21564-17-0]	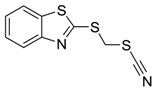	238.34	36.15	3.3 [15]	1: 0.63; 2: 0.41; 3: 2.67; 4: 3.50; 5: 0.09; 6: 0.03; 7: 0.48; A: NO B: NO	F: 19.40 (28.8) D: 12.21 A: 13.94	
Diuron [330-54-1]	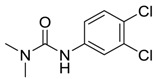	233.09	32.34	2.8 [15]	1: 0.27; 2: 0.01; 3: 2.27; 4: 3.18; 5: 0.06; 6: 0.01; 7: −0.41; A: NO B: NO	F: 47.65 (0.53) D: 28.79 (7.7) A: 27.73 (9.32)	
DCOIT [64359-81-5]	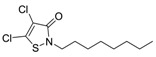	282.22	20.31	2.8 [15]	1: 0.50; 2: 0.05; 3: 2.53; 4: 3.51; 5: 0.22; 6: 0.02; 7: 0.59; A: NO B: NO	F: 8.65 D: 5.69 A: 7.78
Zinc pyrithione (Zinc omadine) [13463-41-7]	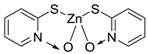	317.69	23.47 **	0.9 [15]	1: 0.69 **; 2: 0.77 **; 3: 2.92 **; 4: 3.66 **; 5: 0.36 **; 6: 0.26 **; 7: 0.54 **; A: YES B: NO **	F: 12190 ** (0.003) D: 5596 ** (0.008) A: 1731 **
Copper pyrithione (Copper omadine) [14915-37-8] or [154592-20-8]	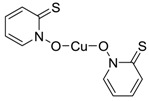	315.85	23.47 **	−0.3 **	1: 0.69 **; 2: 0.77 **; 3: 2.92 **; 4: 3.66 **; 5: 0.36 **; 6: 0.26 **; 7: 0.54 **; A: YES B: NO **	F: 12190 ** D: 5596 ** A: 1731 ** (0.035)
Zineb [12122-67-7]	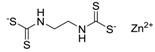	275.73	24.06 **	0.8	1: 0.64 **; 2: 0.50 **; 3: 2.73 **; 4: 3.54 **; 5: 0.18 **; 6: 0.06 **; 7: 0.89 **; A: NO B: NO **	F: 3036 ** (>180) D: 1517 ** (0.6–1.8) A: 667 **

In contrast to the predicted aerobic biodegradation as derived from BIOWIN™ calculations whereby all biocides are not readily biodegraded, experimental degradation data [2,16,17] suggest that only Irgarol 1051 and Diuron are not readily biodegradable (Table 2). These two compounds have also been detected in waters that are linked to vessel activity [15,18]. Studies have shown that the half-life of Irgarol 1051 is between 100–350 days in seawater and several of the degradation products are more toxic and persistent than the parent compound [7,19,20]. Diuron is also reported to be persistent in seawater [21]. In contrast, DCOIT, which won the Presidential Green Chemistry award of the U.S. EPA, has a half-life of <1–24 h in both aerobic and anaerobic aquatic microcosms and is considered a low environmental risk [6,21]. Table 2 summarizes the predicted biodegradation (BIOWIN™ 3 and 4 respectively for aerobic conditions and BIOWIN™ 7 for anaerobic conditions) in comparison with the measured half-lives. Wherever possible, the data for biodegradability are selected from tests where the inoculum is from water (aerobic) or sediment (anaerobic) sources. Under anaerobic biodegradation conditions, both predicted and experimental data for all compounds are in agreement except Chlorothalonil. As already noted, the measured values depend on the conditions of study and hence are not reproducible but could nevertheless provide a reasonable estimate of biodegradability. Caution must be taken for the prediction of copper/zinc pyrithione as calculations are for the ligand without the metal.

Experimentally-obtained values for biodegradability differed significantly from the values predicted by BIOWIN™ (Table 2). This is not unexpected given the limited datasets used in the development of BIOWIN™, and the fact that models cannot make good predictions for structures that they do not take into account. Models for BIOWIN™ 1 and 2 were derived from a dataset of approximately 295 chemicals to derive the fragment coefficients, of which 186 compounds were critically evaluated as “biodegrades fast” and 109 chemicals evaluated as “does not biodegrade fast” [22]. BIOWIN™ 3 and 4 were derived from the evaluation of 200 compounds by an expert panel conducted by the EPA, whilst the Japanese MITI BIOWIN™ 5 and 6 developed their models based on 385 chemicals that were critically evaluated as “readily degradable” and 499 chemicals that were critically evaluated as not readily biodegradable”. In general, the biodegradability predictions were useful for most compounds. Compounds that have been detected in the waterways are clearly not readily biodegradable and these are predicted correctly from BIOWIN™. However it should be noted that the converse may not true, *i.e*., not all compounds that are predicted to be not readily biodegradable are experimentally observed to be so. The veracity of the latter statement cannot easily be ascertained as experimental half-life measurements may not reflect “true” field conditions, and the absence of any substance in the waterways may be due to sampling and monitoring methods utilized. In addition, one would also need to consider the volume of the chemicals in question as typically compounds can only be detected when the market share increases.

#### 2.1.1.1. Metabolites

**Figure 1:**
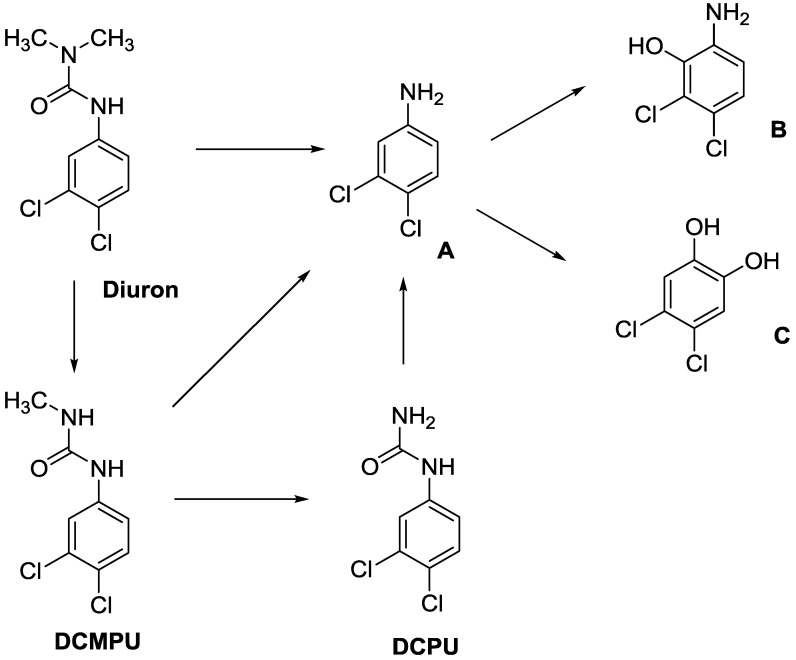
Some of the predicted metabolites from the biodegradation of Diuron.

In principle, ultimate degradation is the true measure of biodegradation as it is possible that metabolites from primary and subsequent degradation(s) are PBT compounds. Biodegradation pathways can be predicted using University of Minnesota Biocatalysis/Biodegradation Database (UM-BBD, http//umbbd.msi.umn.edu/index.html) and the metabolites recalculated for biodegradability using BIOWIN™. This is illustrated with Diuron as shown in Figure 1. Some of these metabolites (DCMPU and DCPU) have been detected experimentally [23].

Physicochemical properties and BIOWIN™ calculations of the predicted metabolites of Diuron are summarized in Table 3. In the case of the metabolites of Diuron, the predicted biodegradation of the metabolites are similar to that of the parent compound, which render them not readily biodegradable.

**Table 2:** Comparison of predicted biodegradation using BIOWIN™ 3, 4 and 7 with experimental observations. For predicted biodegradation from BIOWIN™ 3 and 4, timescale for degradation was obtained from BIOWIN™ and broadly corresponds to values of >5 = hours, >4 = days, >2 = months, <2 = recalcitrant. * Prediction for the ligand only. For predicted probability of fast biodegradation from BIOWIN™ 7, values <0.5 are considered to be not readily biodegradable (N). For experimental data, we define < 24 h = hours, 24 h < days < 168 h/7 days, 168 h/7 days < weeks < 30 days, months > 30 days.

Booster biocides	Aerobic conditions				Anaerobic conditions	
	BIOWIN™ 3-predicted time for ultimate biodegradation	BIOWIN™ 4-predicted time for primary biodegradation	Experimental half-life [6,16,17,21]	Predicted readily biodegradable (Y) or not readily biodegradable (N)	BIOWIN™ 7-predicted probability for fast biodegradation Fast (Y) or Slow (N)	Experimental half-life [6,16,17,21]
Chlorothalonil	Recalcitrant	Weeks to months	Hours to days	N	N	Hours
Dichlofluanid	Months	Weeks	Hours	N	N	Hours to days
TCMTB	Weeks to months	Days to weeks	Days	N	N	Days
Diuron	Weeks to months	Weeks	Months	N	N	Weeks
DCOIT	Weeks to months	Days to weeks	Hours	N	Y	Hours
Irgarol 1051	Weeks to months	Days to weeks	Months	N	N	Persistent
Zinc/copper pyrithione	Weeks *	Days to weeks *	Hours	N *	Y *	Hours

**Table 3:** BIOWIN™ calculations for Diuron and metabolites. *M*_r_ = molecular weight; tPSA = total polar surface area; Log *K*_ow_ = log of water/octanol partition coefficient. * Log *K*_ow_ values calculated using KOWWIN. BIOWIN™ models: 1-linear, 2-non-linear, 3-ultimate, 4-primary, 5-linear MITI, 6-non-linear MITI, 7-anaerobic; Criteria A-model 2 or 6 > 0.5 and model 3 > 2.2; Criteria B-model 3 “weeks” or faster and model 5 > 0.5. Letters correspond to structures in Figure 1.

Structure	*M*_r_	tPSA	Log *K*_ow_	BIOWIN™
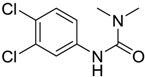 **Diuron**	233.09	32.34	2.68	1: 0.27; 2: 0.01; 3: 2.27; 4: 3.18; 5: 0.05; 6: 0.01; 7: −0.41; A: NO B: NO
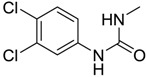 **DCMPU**	219.07	41.13	2.94	1: 0.28; 2: 0.02; 3: 2.30; 4: 3.20; 5: 0.10; 6: 0.02; 7: −0.33; A: NO B: NO
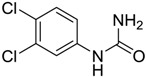 **DCPU**	205.04	55.12	2.65	1: 0.28; 2: 0.02; 3: 2.33; 4: 3.22; 5: 0.14; 6: 0.03; 7: −0.25; A: NO B: NO
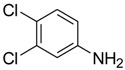 **A**	162.01	26.02	2.69	1: 0.07; 2: 0.01; 3: 2.29; 4: 3.17; 5: 0.11; 6: 0.03; 7: −0.53; A: NO B: NO
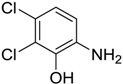 **B**	178.01	46.25	1.88 *	1: 0.18; 2: 0.01; 3: 2.31; 4: 3.19; 5: 0.12; 6: 0.03; 7: −0.36; A: NO B: NO
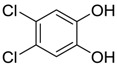 **C**	179.00	40.46	2.32 *	1: 0.53; 2: 0.15; 3: 2.50; 4: 3.34; 5: 0.34; 6: 0.13; 7: 0.002; A: NO B: NO

#### 2.1.1.2. Ecological Effects

Chlorothalonil, Dichlofluanid, Irgarol 1051, TCMTB have log *K*_ow_ values of >3 which implies the potential for bioaccumulation. Bioaccummulation factors (BCF) have been reported on Material Safety Data Sheets (MSDS) for Chlorothalonil (trout), Diuron (mosquito fish) and Zineb (trout) as 436,690, 290 and 770 respectively. ECOSAR™ predicts that the least toxic biocides are pyrithione ligand (metal not included in the calculations) while both Chlorothalonil and DCOIT are amongst the most toxic to aquatic animals (Table 1). MSDS data for the compounds in Table 1 suggest that Chlorothalonil, Dichlorofluanid, Irgarol 1051, Diuron, Zinc Pyrithione and Zineb have hazard warnings of toxicity to aquatic life (H400, H410, Sigma-Aldrich, St. Louis, MO, USA). Other data suggest that DCOIT is acutely toxic to a wide range of aquatic organisms though does not cause chronic toxicities [24]. We note that ECOSAR™ does not perform well in the prediction of acute toxicity towards aquatic organisms. This may be attributed to the differences in study conditions between the experiments and those used in the development of the ECOSAR™ model which focused primarily on the terrestrial freshwater ecosystem.

#### 2.1.2. Pharmaceuticals/Veterinary Medicines as Anti-Fouling Agents

A number of pharmaceuticals and veterinary medicines have been screened for anti-settlement activity [25] and one of these, Medetomidine, is being developed as an anti-foulant [26]. However, pharmaceuticals and veterinary medicines are in general, designed to be stable, both chemically and metabolically—The former to improve the shelf life of the compounds, while the latter to ensure that the drug reaches the desired target(s) for maximal therapeutic effects. The problem of persistence of pharmaceuticals and veterinary medicines in our environment is well-documented and is an area of concern [27].

Table 4 summarises some of the pharmaceuticals and veterinary medicines that have been shown to possess anti-fouling properties. Using criteria A, Sibelium, Zyrtec, Clarityne, Cyclizine, Celebrex, Diclomelan, Prozac, Haldol, Zyprexa, Exelon, Aricept/Donepezic, Imodium, Phentolamine, Prazosin and Clonidine are not readily biodegraded. In contrast, using criteria B, none of the compounds are predicted to be biodegradable. Zyrtec, Clarityne, Dichlomelan, Ponstan, Prozac and Haldol are considered high production volume (HPV) products and have been detected in the environment [11]. In an analysis by Howard and Muir [11] using BIOWIN™ 1 and 5 on the neutral drug, as well as scientific judgment of the chemical structures, Cyclizine, Celebrex, Zyprexa, Phentolamine, Medetomidine, Imodium and Clonidine are estimated to be persistent and bioaccumulative though these have not yet been detected in the environment.

Medetomidine is currently undergoing assessment as part of the biocide product directive (BPD) for use as an anti-fouling biocide, under the product label, Selektope™. The MSDS for this indicates that Medetomidine is highly toxic to aquatic organisms, is not readily biodegradable but it is not expected to bioaccumulate, which contradicts Howard and Muir’s prediction that Medetomidine is expected to bioaccumulate [11]. Table 5 compares the ECOSAR™ predicted toxicity of pharmaceuticals and veterinary medicines against available ecological toxicity data from MSDS. Again, ECOSAR™ does not seem to perform well in the prediction of acute toxicity towards aquatic organisms. ECOSAR™ has been found to perform better on some classes of compounds compared to others [28]. There are several reasons why model predictions may differ from reality. First, in the development of the QSAR model, only experimental data for compounds in the training set under specific study conditions were used. As such, comparison should only be carried out for compounds under similar experimental conditions. Furthermore, the QSAR model calculates toxicity values for new chemicals by inserting the estimated *K*_ow_ into a regression equation and correcting the resultant value for the molecular weight of the compound. However, log *K*_ow_ values cannot be calculated for compounds with metals, coordination bonds and salts and this reduces the accuracy of model predictions. The current status for assessing aquatic toxicity is through professional judgment and experimental data wherever reported.

#### 2.1.3. Miscellaneous

There are a large number of synthetic chemicals with anti-foulant properties that are not known biocides or pharmaceutical compounds. These have varied structures and chemical functionalities and in most cases, are low molecular weight compounds. In our opinion, this is a promising source of environmentally benign chemicals for the development of anti-foulants as it is likely that there are compounds in this category that fulfill the biodegradability requirements and are not too chemically complex in terms of the economics of synthesis. Examples of these compounds include phenyl ethers, diketopiperazines, and butenolides. Should one consider the prediction criteria A to be readily biodegradable then all the compounds except two butenolides listed in Table 6 are readily biodegradable. Using the prediction criteria B, six of the compounds are predicted to be biodegradable. Eleven of the 18 compounds have log *K*_ow_ values of >3 which indicates the potential for bioaccumulation.

**Table 4:** Pharmaceuticals and veterinary medicines possessing anti-fouling properties. EC_50_ = effective concentration at which 50% of larval settlement was prevented; LC_50_ = concentration lethal to 50% of nauplii; *M*_r_ = molecular weight; tPSA = total polar surface area calculated; Log *K*_ow_ = log of water/octanol partition coefficient. Log *K*_ow_ of the ionised or neutral form of pharmaceuticals (as indicated) were calculated. BIOWIN™ models: 1-linear, 2-non-linear, 3-ultimate, 4-primary, 5-linear MITI, 6-non-linear MITI, 7-anaerobic; Criteria A-model 2 or 6 > 0.5 and model 3 > 2.2; Criteria B-model 3 “weeks” or faster and model 5 > 0.5. Biological data for Phentolamine, Prazosin, Medetomidine and Clonidine were obtained from [29]. Biological data for all other compounds were obtained from [25].

Pharmaceuticals/Veterinary medicines	Name [CAS number]	Biological activity		*M*_r_	Log *K*_ow_	tPSA	BIOWIN™
		EC_50_ (Cyprid) µg/mL	LC_50_ (Naupliar) µg/mL				
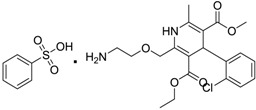	Norvasc [111470-99-6]	0.32	0.71	567.05 (salt) 409.89 (ionised form of drug)	0.14 (ionised form of drug)	155.87 (ionised form of drug)	1: 0.58; 2: 0.83; 3: 2.31; 4: 3.51; 5: 0.43; 6: 0.09; 7: −0.34; A: YES B: NO
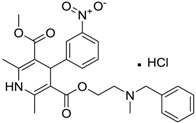	Cardepine/Nicardipine [54527-84-3]	0.18	0.05	515.99 (salt) 480.54 (ionised form of drug)	2.39 (ionised form of drug)	120.88 (ionised form of drug)	1: 0.90; 2: 1.00; 3: 2.21; 4: 3.48; 5: −0.09; 6: 0.001; 7: −0.29; A: YES B: NO
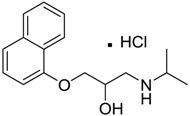	Inderal [318-98-9]	2.5	0.040	295.81 (salt) 260.36 (ionised form of drug)	0.74 (ionised form of drug)	46.07 (ionised form of drug)	1: 0.91; 2: 0.93; 3: 2.72; 4: 3.68; 5: 0.34; 6: 0.29; 7: −0.40; A: YES B: NO
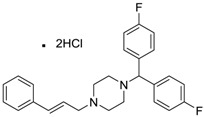	Sibelium [30484-77-6]	0.2	3.4	477.42 (salt) 404.50 (ionised form of drug)	4.91 (ionised form of drug)	8.88 (ionised form of drug)	1: −0.94; 2: 0; 3: 1.50; 4: 3.29; 5: −0.24; 6: 0; 7: −1.19; A: NO B: NO
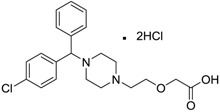	Zyrtec [83881-52-1]	0.04	25	461.81 (salt) 390.91 (ionised form of drug)	2.90 (ionised form of drug)	55.41 (ionised form of drug)	1: 0.23; 2: 0.004; 3: 2.50; 4: 3.50; 5: 0.01; 6: 0.02; 7: −1.08; A: NO B: NO
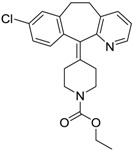	Clarityne/Loratidine [79794-75-5]	0.5	0.62	382.89 (neutral)	5.20 (neutral)	41.9 (neutral)	1: 0.42; 2: 0.02; 3: 1.74; 4: 3.17; 5: −0.43; 6: 0.001; 7: −0.27; A: NO B: NO
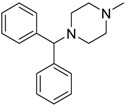	Cyclizine [82-92-8]	0.04	0.04	266.39 (neutral)	2.97 (neutral)	6.48 (neutral)	1: 0.47; 2: 0.16; 3: 2.14; 4: 2.90; 5: −0.10; 6: 0.01; 7: −2.36; A: NO B: NO
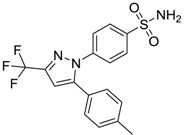	Celebrex/Celecoxib [169590-42-5]	4	4	381.37 (neutral)	3.47 (neutral)	75.76 (neutral)	1: 0.10; 2: 0.0006; 3: 1.77; 4: 2.95; 5: −0.25; 6: 0.00; 7: −0.39; A: NO B: NO
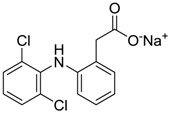	Diclomelan/Diclofenac [15307-79-6]	0.2	1.6	318.13 (salt) 295.14 (ionised form of drug)	4.51 (ionised form of drug)	52.16 (ionised form of drug)	1: 0.13; 2: 0.003; 3: 2.29; 4: 3.30; 5: −0.13; 6: 0.003; 7: −0.85; A: NO B: NO
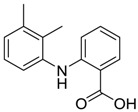	Ponstan/Mefenamic acid[61-68-7]	0.2	5.2	241.29 (neutral)	5.12 (neutral)	49.33 (neutral)	1: 0.67; 2: 0.78; 3: 2.47; 4: 3.26; 5: 0.35; 6: 0.15; 7: −0.59; A: YES B: NO
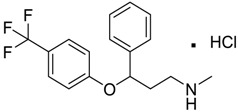	Prozac/Fluoxetine [56296-78-7]	1.5	0.2	345.79 (salt) 310.34 (ionised form of drug)	2.79 (ionised form of drug)	25.84 (ionised form of drug)	1: 0.34; 2: 0.05; 3: 1.96; 4: 3.21; 5: 0.19; 6: 0; 7: −0.06; A: NO B: NO
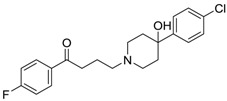	Haldol/Haloperidol [52-86-8]	<0.04	0.26	375.87 (neutral)	4.30 (neutral)	40.54 (neutral)	1: −0.81; 2: 0; 3: 1.27; 4: 2.69; 5: 0.01; 6: 0.0001; 7: −2.53; A: NO B: NO
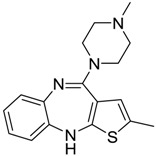	Zyprexa/Olanzapine [132539-06-1]	0.04	0.2	312.44 (neutral)	3.00 (neutral)	30.87 (neutral)	1: 0.21; 2: 0.007; 3: 2.04; 4: 2.93; 5: −0.30; 6: 0.001; 7: −1.92; A: NO B: NO	
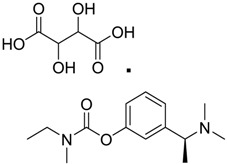	Exelon/Rivastigmine [129101-54-8]	25	0.026	400.43 (salt) 252.36 (ionised form of drug)	1.31 (ionised form of drug)	35.18 (ionised form of drug)	1: 0.63; 2: 0.35; 3: 2.64; 4: 3.48; 5: 0.02; 6: 0.04; 7: −0.32; A: NO B: NO	
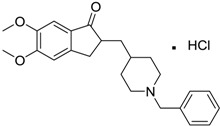	Aricept/Donepezic [884740-09-4]	0.28	0.52	415.96 (salt) 380.51 (ionised form of drug)	3.35 (ionised form of drug)	39.97 (ionised form of drug)	1: 1.02; 2: 0.98; 3: 2.16; 4: 3.37; 5: 0.18; 6: 0.05; 7: −0.75; A: NO B: NO	
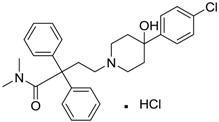	Imodium/Loperamide [34552-83-5]	>5	0.24	513.50 (salt) 478.05 (ionised form of drug)	3.63 (ionised form of drug)	44.98 (ionised form of drug)	1: 0.44; 2: 0.05; 3: 1. 50; 4: 2.90; 5: −0.16; 6: 0.002; 7: −2.73; A: NO B: NO	
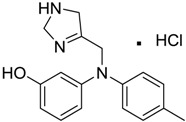	Phentolamine [73-05-2]	MIC 0.28 MTC 28.1	317.82 (salt) 282.37 (ionised form of drug)	2.10 (ionised form of drug)	52.44 (ionised form of drug)	1: 0.58; 2: 0.15; 3: 2.30; 4: 3.12; 5: 0.04; 6: 0.03; 7: −1.95; A: NO B: NO	
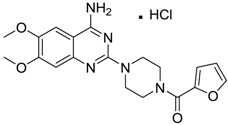	Prazosin [19237-84-4]	MIC 0.38 MTC 1.53	419.87 (salt) 384.42 (ionised form of drug)	2.16 (ionised form of drug)	103.6 (ionised form of drug)	1: 0.83; 2: 0.92; 3: 1.92; 4: 3.36; 5: 0.11; 6: 0.02; 7: −2.00; A: NO B: NO	
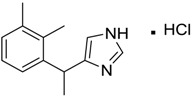	Medetomidine [145108-58-3]	MIC 0.0002 MTC 20.029	236.74 (salt) 201.29 (ionised form of drug)	−0.15 (ionised form of drug)	28.97 (ionised form of drug)	1: 0.82; 2: 0.87; 3: 2.53; 4: 3.35; 5: 0.23; 6: 0.14; 7: −0.55; A: YES B: NO	
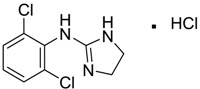	Clonidine [4205-91-8]	MIC 0.00023 MTC 34.51	266.55 (salt) 231.10 (ionised form of drug)	0 (ionised form of drug)	41 (ionised form of drug)	1: 0.04; 2: 0.002; 3: 2.14; 4: 3.07; 5: −0.07; 6: 0.009; 7: −1.21; A: NO B: NO	

**Table 5:** Comparison of predicted ECOSAR™ toxicity against Material Safety Data Sheets (MSDS) data for selected pharmaceuticals and veterinary medicines.

Structure	Name [CAS number]	ECOSAR™ acute toxicity (mg/L)			MSDS ecological toxicity (mg/L)		
		Fish LC_50_	Daphnid EC_50_	Green algae EC_50_	Fish LC_50_	Daphnid EC_50_	Green algae EC_50_
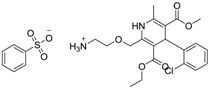	Norvasc [111470-99-6]	15,904	7602	2779	14	9.9	0.28
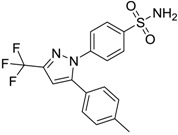	Celebrex/Celecoxib [169590-42-5]	14.858	9.667	12.642	1.2	1.5	–
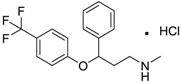	Prozac/Fluoxetine [56296-78-7]	49.31	30.13	30.39	1.57	0.94	–
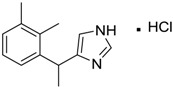	Medetomidine [145108-58-3]	14,042.98	6539.26	2145.39	30	4.5	0.34

**Table 6:** Synthetic compounds of *M*_r_ < 400 with anti-fouling properties. EC_50_ = effective concentration at which 50% of larval settlement was prevented; *M*_r_ = molecular weight; tPSA = total polar surface area; Log *K*_ow_ = log of water/octanol partition coefficient. All log *K*_ow_ values were calculated using KOWWIN™. BIOWIN™ models: 1-linear, 2-non-linear, 3-ultimate, 4-primary, 5-linear MITI, 6-non-linear MITI, 7-anaerobic; Criteria A-model 2 or 6 > 0.5 and model 3 > 2.2; Criteria B-model 3 “weeks” or faster and model 5 > 0.5. ECOSAR™: F = fish, D = daphnid, A = green algae.

Class of compound	Structure	Antifouling activity EC_50_ (Cyprid) µg/mL [REF]	*M*_r_	Log *K*_ow_	tPSA	BIOWIN™	ECOSAR™ acute toxicity (mg/L)	
Diphenyl ethers	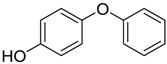	0.81 [30]	186.21	3.35	29.46	1: 1.05; 2: 1.00; 3: 2.82; 4: 3.70; 5: 0.49; 6: 0.51; 7: 0.45; A: YES B: NO	F: 5.96 D: 3.91 A: 5.31	
Diphenyl ethers	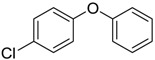	0.31 [30]	204.65	4.70	9.23	1: 0.73; 2: 0.89; 3: 2.50; 4: 3.47; 5: 0.39; 6: 0.23; 7: −0.03; A: YES B: NO	F: 0.64 D: 0.47 A: 0.97	
Diketopiperazines	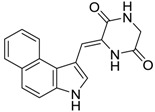	1.5 [26]	291.31	1.20	70.23	1: 1.03; 2: 1.00; 3: 2.45; 4: 3.84; 5: 0.18; 6: 0.05; 7: −1.16; A: YES B: NO	F: 1252.82 D: 660.67 A: 362.55	
Diketopiperazines	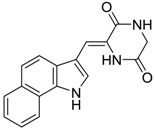	0.034 [26]	291.31	1.20	70.23	1: 1.03; 2: 0.99; 3: 2.45; 4: 3.84; 5: 0.18; 6: 0.05; 7: −1.16; A: YES B: NO	F: 1252.82 D: 660.67 A: 362.55	
Butenolides	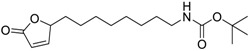	0.69 [31]	311.42	3.82	64.63	1: 0.67; 2: 0.88; 3: 2.39; 4: 3.67; 5: 0.57; 6: 0.55; 7: 0.53; A: YES B: NO	F: 5.94 D: 3.99 A: 5.95	
Butenolides	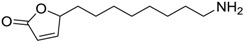	7.62 [31]	211.31	1.86	52.32	1: 0.97; 2: 0.99; 3: 2.90; 4: 3.82; 5: 0.88; 6: 0.88; 7: 1.29; A: YES B: YES	F: 230.25 D: 129.10 A: 91.28
Butenolides	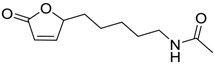	3.55 [31]	211.26	0.33	55.4	1: 1.03; 2: 0.99; 3: 2.82; 4: 4.00; 5: 0.83; 6: 0.86; 7: 0.38; A: YES B: YES	F: 5458.77 D: 2657.20 A: 1047.22
Butenolides	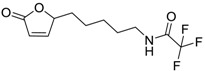	2.39 [31]	265.23	1.38	55.4	1: 0.49; 2: 0.59; 3: 2.19; 4: 3.63; 5: 0.72; 6: 0; 7: 0.46; A: NO B: NO	F: 777.89 D: 417.29 A: 245.76
Butenolides	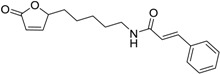	1.22 [31]	299.37	2.32	55.4	1: 1.12; 2: 1.00; 3: 2.65; 4: 3.86; 5: 0.62; 6: 0.49; 7: 0.06; A: YES B: NO	F: 127.86 D: 74.75 A: 62.83
Butenolides	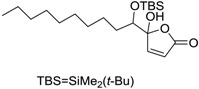	1.5 [32]	370.60	7.02	55.76	1: 0.49; 2: 0.56; 3: 2.39; 4: 3.50; 5: 0.45; 6: 0.19; 7: −0.56; A: YES; B: NO	F: 0.01 D: 0.009 A: 0.043
Butenolides	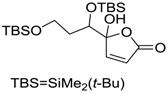	3.0 [32]	402.67	5.95	64.99	1: 0.18; 2: 0.02; 3: 1.81; 4: 3.03; 5: 0.13; 6: 0.01; 7: −1.05; A: NO B: NO	F: 0.094 D: 0.077 A: 0.258
Butenolides	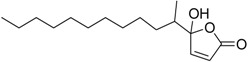	3.0 [32]	268.39	5.63	46.53	1: 0.72; 2: 0.97; 3: 2.83; 4: 3.81; 5: 0.73; 6: 0.82; 7: 0.12; A: YES B: YES	F: 0.122 D: 0.097 A: 0.288
Phenyl ethers	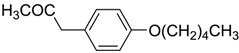	10 [33]	220.31	3.52	26.3	1: 0.94; 2: 0.98; 3: 2.85; 4: 3.80; 5: 0.55; 6: 0.61; 7: −0.25; A: YES B: YES	F: 7.84 D: 5.1 A: 6.81
Phenyl ethers	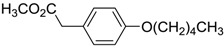	10.4 [33]	236.31	4.13	35.53	1: 1.10; 2: 1.00; 3: 2.98; 4: 4.01; 5: 0.72; 6: 0.83; 7: 0.31; A: YES B: YES	F: 2.39 D: 1.65 A: 2.77	
Pyridyl ethers	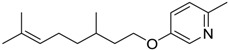	0.02 [34]	247.38	5.62	21.59	1: 0.66; 2: 0.66; 3: 2.31; 4: 3.48; 5: 0.36; 6: 0.20; 7: 0.62; A: YES B: NO	F: 0.11 D: 0.09 A: 0.27	
Juvenoid	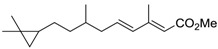	0.01 [34]	264.41	6.50	26.3	1: 0.61; 2: 0.83; 3: 2.54; 4: 3.54; 5: 0.49; 6: 0.31; 7: −0.11; A: YES B: NO	F: 0.02 D: 0.02 A: 0.07	
Capsaicin derivative	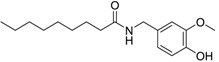	5.19 [35]	293.41	3.79	58.56	1: 1.17; 2: 1.00; 3: 2.79; 4: 4.02; 5: 0.54; 6: 0.51; 7: −0.06; A: YES B: YES	F: 5.90 D: 3.95 A: 5.84	
*N*-benzoyl monoethanolamine benzoate	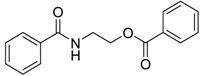	6.92 [35]	269.30	2.69	55.4	1: 1.26; 2: 1.00; 3: 2.73; 4: 3.90; 5: 0.56; 6: 0.54; 7: −0.03; A: YES B: NO	F: 53.02 D: 32.08 A: 31.12	

### 2.2. Natural Products as Anti-Foulants

**Figure 2:**
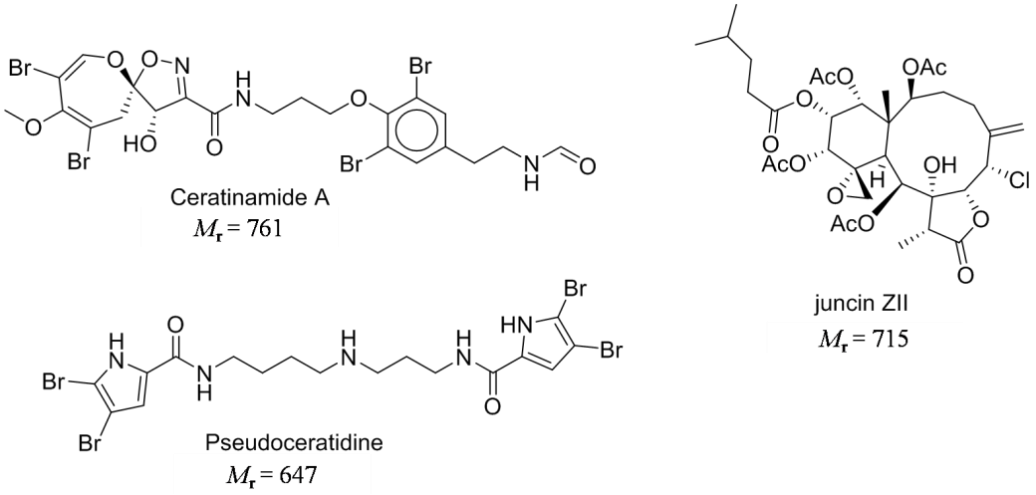
Examples of complex natural products containing anti-foulant activities.

Natural products are organic compounds produced by living organisms. Those produced as secondary metabolites usually show biological activities as they are not essential for life but provide evolutionary advantages e.g., chemical defense against predators. Natural products have rich structural diversities and more often than not, possess multiple functionalities and have relatively high molecular weights. The role and importance of natural products in drug discovery and development have long been recognised [36,37]. Natural products have also been tested for anti-fouling activities and a number of these compounds have shown promising anti-settlement activities against fouling organisms [38,39]. When considering the development of compounds for antifouling management, are natural products necessarily better? For large complex natural products, the answer is clearly no. The economics of production would dictate that any product for anti-fouling management should be cheap (and hence easy) to make. The total syntheses of large complex natural products do not fulfil this criteria and any large scale production will not be sustainable. From a practical viewpoint, small natural products (defined as *M*_r_ < 400) show more promise for development as anti-foulants. The assumption has been that as natural products have existed for millions of years, the bacteria responsible for their degradation would very likely have co-evolved, and thus they would likely be biodegradable. However, as shown in Figure 2, some natural products possess functionalities that suggest that biodegradation will be slow. For example, ceratinamide A and pseudoceratidine are highly brominated while juncin ZII have multiple quaternary carbon centres. Thus considerations of natural products as potential “green” anti-foulants cannot be simplistically argued as “natural is safe”.

Molecular complexity is retained by some of the “small” natural products as indicated by their multiple functional groups and stereochemistries. Under criteria A, eight out of the 17 listed compounds are predicted to be readily biodegradable. In contrast, under prediction criteria B, none of the compounds are predicted to be readily biodegradable. Twelve out of seventeen compounds have log *K*_ow_ values of >3, which implies the potential for bioaccumulation.

The structurally “less complex” natural products in the Table such as isocyanotheonellin, moloka’iamine A, 5,4'-dihydroxy-7-trimethoxyflavone, 3-chloro-2,5-dihydroxybenzyl alcohol, brominated furanone, and capsaicin are worthy of further considerations as these are synthetically accessible. In some cases, structure-activity relationships of the natural products have been studied and synthetic analogues synthesised and evaluated as anti-foulants. Of note from the natural products listed in Table 7 are capsaicin and brominated furanone. The former is considered an “emerging” anti-foulant and recent biodegradation studies conclude that capsaicin is readily biodegradable, has a low potential to bioaccumulate and is therefore a low environmental risk [40].

In natural product drug development, the pharmacophore is retained in the design of the drug and functionalities are added to this pharmacophore to improve potency and drug-like properties. This “nature-inspired” approach can also be utilized in the development of new anti-foulants. The analysis of the natural products in Table 7 suggests potential structural classes of compounds for anti-settlement activities. These include the alkyl isocyanides, flavones, and furanones. The brominated furanone, one of the furanone natural products isolated from the Australian red alga *Delisa pulchra*, shows potent antifouling activity against *Amphibalanus amphitrite* cyprids as well as inhibits the settlement and growth of algal gametes and bacteria [41]. Extensive structure-activity studies have been carried out on the furanones and a recent publication by Qian *et al.* on furanones demonstrate the relationship between bioactivity of the compounds with physicochemical parameters such as lipophilicity [31]. Despite these advances in anti-fouling research, we note that there have been no deliberate attempts to “design in” features to improve biodegradation [42,43] and reduce toxicities. A further consideration, one that is not well-understood yet, is to ensure that the designed compounds retain the physicochemical properties for easy formulation into anti-fouling coatings. The analogy for this in medicinal chemistry is that any drug developed for oral intake should conform to the rule of 5 (Lipinski’s rules) [44,45]. To our knowledge, there are no such published guidelines for organic compounds developed as additives into anti-fouling paints or coatings.

**Table 7:** Natural products, *M*_r_ < 400, with anti-fouling properties. EC_50_ = effective concentration at which 50% of larval settlement was prevented; *M*_r_ = molecular weight; tPSA = total polar surface area; Log *K*_ow_ = log of water/octanol partition coefficient. All log *K*_ow_ values were calculated using KOWWIN™. BIOWIN™ models: 1-linear, 2-non-linear, 3-ultimate, 4-primary, 5-linear MITI, 6-non-linear MITI, 7-anaerobic; Criteria A-model 2 or 6 > 0.5 and model 3 > 2.2; Criteria B-model 3 “weeks” or faster and model 5 > 0.5. ECOSAR™: F = fish, D = daphnid, A = green algae.

Class of compound	Structure	Name	Antifouling activity EC_50_ (Cyprid) µg/mL [REF]	*M*_r_	Log *K*_ow_	tPSA	BIOWIN™	ECOSAR™ acute toxicity (mg/L)
Diterpenoids	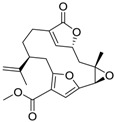	Pukalide	0.019 [46]	372.42	3.67	74.36	1: 0.44; 2: 0.77; 3: 2.36; 4: 3.54; 5: 0.41; 6: 0.10; 7: −0.34; A: YES B: NO	F: 9.66 D: 6.40 A: 9.02
Diterpenoids	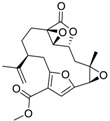	Epoxypukalide	0.055 [46]	388.42	3.28	86.89	1: −0.10; 2: 0.02; 3: 2.10; 4: 3.35; 5: 0.44; 6: 0.07; 7: −0.81; A: NO B: NO	F: 22.79 D: 14.56 A: 17.65
Terpenoids	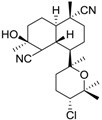	Kalihinol A	0.087 [47]	392.97	3.91	77.04	1: −0.02; 2: 0.004; 3: 1.14; 4: 2.43; 5: 0.14; 6: 0.001; 7: −1.69; A: NO B: NO	F: 6.26 D: 4.24 A: 6.54
Terpene	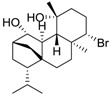	Bromo tetrasphaerol	0.38 [48]	385.39	5.32	40.46	1: 0.13; 2: 0; 3: 1.90; 4: 3.00; 5: 0.17; 6: 0.002; 7: −0.64; A: NO B: NO	F: 0.33 D: 0.26 A: 0.68
Sesquiterpene carbonimide dichloride	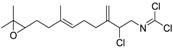	Axinyssimide A	1.2 [49]	352.72	7.63	24.89	1: −0.29; 2: 0; 3: 1.68; 4: 2.87; 5: −0.04; 6: 0.0007; 7: −0.25; A: NO B: NO	F: 0.003 D: 0.002 A: 0.02
Isocyano-sesquiterpene	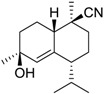	*(1S, 4R, 7S, 10R)-*10-Isocyano-5-cadinen-4-ol	0.17 [49]	247.38	3.75	44.02	1: 0.57; 2: 0.67; 3: 2.15; 4: 3.12; 5: 0.24; 6: 0.05; 7: −0.79; A: NO B: NO	F: 5.40 D: 3.60 A: 5.25
Sesquiterpene hydroquinone	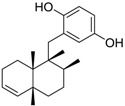	Avarol	0.65 [50]	314.47	6.92	40.46	1: 0.33; 2: 0.01; 3: 1.90; 4: 2.94; 5: 0.18; 6: 0.04; 7: −1.32; A: NO B: NO	F: 0.010 D: 0.009 A: 0.04
Sesquiterpene	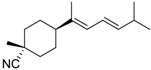	3-isocyanotheonellin	0.13 [51]	231.38	5.99	23.79	1: 0.77; 2: 0.93; 3: 2.39; 4: 3.30; 5: 0.22; 6: 0.07; 7: −0.64; A: YES B: NO	F: 0.05 D: 0.04 A: 0.14
Bromotyramine	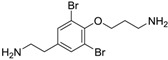	Moloka’iamine A	4.3 [52]	352.07	2.71	61.27	1: 0.85; 2: 0.43; 3: 2.07; 4: 3.13; 5: 0.42; 6: 0.11; 7: 1.16; A: NO B: NO	F: 66.63 D: 40.40 A: 39.46		
Polyketide	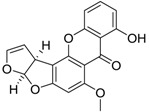	Sterigmatocystin	<0.125 [53]	324.29	4.15	74.22	1: 0.82; 2: 0.94; 3: 2.26; 4: 3.55; 5: 0.56; 6: 0.28; 7: 0.15; A: YES B: NO	F: 3.13 D: 2.17 A: 3.67
Polyacetylene	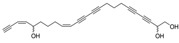	Callytriol C	0.24 [50]	348.44	4.50	60.69	1: 1.06; 2: 0.80; 3: 2.91; 4: 3.73; 5: 0.43; 6: 0.17; 7: 0.79; A: YES B: NO	F: 1.61 D: 1.15 A: 2.23
Flavone	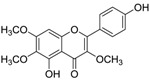	5,4'-dihydroxy-3,6,7-trimethoxy flavone	2.5 [54]	344.32	2.00	94.45	1: 0.87; 2: 0.94; 3: 2.35; 4: 3.63; 5: 0.56; 6: 0.25; 7: 0.17; A: YES B: NO	F: 280.30 D: 159.23 A: 118.80	
Polyhydroxyl benzylalcohol	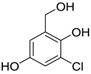	3-chloro-2,5-dihydroxybenzyl alcohol	3.19–3.81 [55]	174.58	0.76	60.69	1: 0.87; 2: 0.81; 3: 2.88; 4: 3.64; 5: 0.45; 6: 0.35; 7: 0.53; A: YES B: NO	F: 1864.70 D: 944.21 A: 438.04	
Aaptamine	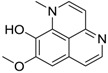	Isoaaptamine	2.65 [56]	228.25	1.88	45.06	1: 0.68; 2: 0.67; 3: 2.44; 4: 3.35; 5: 0.25; 6: 0.07; 7: −0.57; A: YES B: NO	F: 237.95 D: 133.68 A: 95.29	
Butenolide	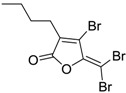	Brominated furanone	0.02 [41]	388.88	2.96	26.3	1: 0.71; 2: 0; 3: 2.87; 4: 3.89; 5: 0.34; 6: 0.001; 7: 1.77; A: NO B: NO	F: 44.27 D: 27.46 A: 29.46	
Capsaicin	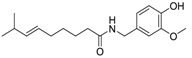	Capsaicin	4.18 [35]	305.42	4.00	58.56	1: 1.06; 2: 0.99; 3: 2.47; 4: 3.73; 5: 0.32; 6: 0.16; 7: −0.21; A: YES B: NO	F: 4.04 D: 2.76 A: 4.40	
Miscellaneous	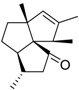	Subergorgic acid	1.2 [57]	218.34	4.38	17.07	1: 0.28; 2: 0.02; 3: 2.27; 4: 3.207; 5: 0.42; 6: 0.15; 7: −0.86; A: NO B: NO	F: 1.31 D: 0.93 A: 1.72	

## 3. Experimental Section

Total polar surface area (tPSA) for all compounds were calculated with Chem Draw. For tPSA calculations of compounds with metals or coordination bonds, the ligand forms without the metals were used.

QSAR prediction for biodegradability was calculated with the Biodegradation Probability Program (BIOWIN™ v4.10, http://www.epa.gov/oppt/exposure/pubs/episuite.htm) developed by Syracuse Research Cooperation jointly with the U.S. EPA. BIOWIN™ utilizes functional group contribution approaches in the estimation of aerobic biodegradation of organic compounds in the presence of microorganisms in the environment. It remains one of the most commonly used free software in the registration process for biocides. For predicted biodegradation from BIOWIN™ 3 and 4, timescale for degradation was obtained from BIOWIN™ and broadly corresponds to values of >5 = hours, >4 = days, >2 = months, <2 = recalcitrant. For predicted probability of fast biodegradation from BIOWIN™ 7, values <0.5 were considered to be not readily biodegradable (N). Experimental half-life values were obtained from the literature or MSDS. For experimental data, we defined <24 h = hours, 24 h < days < 168 h/7 days, 168 h/7 days < weeks < 30 days, months > 30 days.

KOWWIN™ v1.68 (the Log Octanol-Water Partition Coefficient Program) developed by the U.S. EPA was used to estimate the log octanol-water partition coefficient log *K*_ow_, whenever literature or MSDS data are not available. KOWWIN™ uses a “fragment constant” methodology to predict log *P*. In a “fragment constant” method, a structure was divided into fragments (atom or larger functional groups) and coefficient values of each fragment or group were summed together to yield the log *P* estimate. Compounds were considered potentially bioaccumulative if the log *K*_ow_ was 3 or more.

The Ecological Structure Activity Relationships (ECOSAR™) Class Program v1.11 (U.S. EPA, Washington, DC, USA) was used to predict each chemical’s acute (short-term) toxicity and chronic (long-term or delayed) toxicity to aquatic organisms such as fish, aquatic invertebrates, and aquatic plants. Predicted acute toxicity of compounds was compared against the literature or MSDS toxicity data whenever available.

Biodegradation pathway of diuron was predicted using the University of Minnesota Biocatalysis/Biodegradation Database (UM-BBD, http://umbbd.msi.umn.edu/index.html).

The list of pharmaceuticals medicines and its biological data in Table 4 was obtained from a study published by Rittschof *et. al.* [25] and Dahlström *et al.* [29]*.* The tPSA, log *K*_ow_ and QSAR calculations of pharmaceuticals were obtained based on their neutral or ionized forms, as specified.

The lists of synthetic compounds and natural products in Table 6 and Table 7 respectively, were obtained by compiling a few examples from each class of bioactive compounds reported in the literature with low molecular weight (<400) and EC_50_ values <25 µg/mL. The tPSA, log *K*_ow_ and QSAR calculations of these compounds were obtained based on the chemical forms as drawn.

## 4. Conclusions

The study above highlights limitations to the methods of estimation for biodegradation and ecotoxicity predictions. These limitations arise from the limited database as well as the variations in experimental data due to different experimental conditions, design and so on. Despite this, we advocate that the use of these freely available software, especially BIOWIN™, remains valuable as they facilitate the initial selection of compounds for further development given the cost and difficulty of biodegradation assays. Furthermore, biodegradability predictions are important as rapid biodegradability will mitigate any predicted ecological effects of compounds. We suggest that compounds that pass either Criteria A or B have the potential to be biodegradable and could be considered further for development into antifouling coatings. As in drug discovery and development, computational methods cannot totally replace experimental data and validation based on predictions will still need to be carried out. Nevertheless these *in silico* screening tools for the search for “environmentally-benign” antifouling biocides provide a valuable resource in early decision-making when applied with discretion.

We calculated PBT properties for 71 antifouling compounds and metabolites, of which 31 compounds were predicted to be readily biodegradable by either prediction criteria A or B or both by BIOWIN™, criteria B being more stringent than criteria A. Most known biocides and pharmaceuticals were predicted to be not biodegradable. Interestingly, under criteria A, all but two of the miscellaneous synthetic compounds and 53% of the natural products reviewed were predicted to be biodegradable. This result is promising and suggests the potential of low molecular weight (<400) natural products and their analogues for development as “green” anti-foulants. Full PBT experiments with these compounds would be helpful for the development of guidelines to identify potential “green” anti-foulants. In addition, experimental results will allow for validation of the accuracy of predictions by BIOWIN™ and perhaps, ECOSAR™.

The era of environmentally benign anti-foulants is not as yet within our grasp. Although we have been able to identify small synthetic compounds and natural products as a promising source of green antifoulants, there are a number of issues that still need to be addressed for implementing PBT screenings in the development of anti-foulants. Empirical studies are needed to validate current models for predicting PBT. New robust, rapid methods for determining or predicting the PBT properties of compounds early in development are needed.
